# Efficient Protocol for Improving the Development of Cryopreserved Embryonic Axes of Chestnut (*Castanea sativa* Mill.) by Encapsulation–Vitrification

**DOI:** 10.3390/plants10020231

**Published:** 2021-01-25

**Authors:** Mariam Gaidamashvili, Eka Khurtsidze, Tamari Kutchava, Maurizio Lambardi, Carla Benelli

**Affiliations:** 1 Department of Biology, Faculty of Exact and Natural Sciences, Iv. Javakhishvili Tbilisi State University, 1, Chavchavadze Ave., 0179 Tbilisi, Georgia; eka.khurtsidze@tsu.ge (E.K.); tamari.kutchava2013@ens.tsu.edu.ge (T.K.); 2 Institute of BioEconomy, National Research Council (CNR/IBE), Sesto Fiorentino, 50019 Florence, Italy; maurizio.lambardi@ibe.cnr.it (M.L.); carla.benelli@ibe.cnr.it (C.B.)

**Keywords:** activated charcoal, alginate, cryopreservation, European chestnut, zygotic embryo

## Abstract

An optimized cryopreservation protocol for embryonic axes (EAs) of chestnut (*Castanea sativa* Mill.) has been developed based on the encapsulation–vitrification procedure. EAs of mature seeds were aseptically dissected and encapsulated in alginate beads with or without 0.3% (*w*/*v*) activated charcoal (AC). Embedded EAs were dehydrated with Plant Vitrification Solution 2 for different treatment times up to 120 min, followed by direct immersion in liquid nitrogen. Cryopreserved embryonic axes encapsulated with AC showed higher survival (70%) compared to those encapsulated without AC (50%). Sixty-four percent of embryonic axes, from synthetic seeds with AC, subsequently developed as whole plants. Plantlet regrowth was faster in AC-encapsulated EAs and showed enhanced postcryopreservation shoot and root regrowth over 2 cm after five weeks from rewarming. Results indicate that encapsulation–vitrification with activated charcoal added to the beads is an effective method for the long-term preservation of *Castanea* *sativa* embryonic axes.

## 1. Introduction

European chestnut or sweet chestnut (*Castanea sativa* Mill.), belonging to the genus *Castanea,* is dominant in the mountainous forests of Western Georgia (150–1800 m), occupying the highest percentage of areas covered with forests (approx. 75%). Chestnut forests are developed in both West and East Georgia, but to the West of the country, they occupy larger areas. Chestnut trees generally extend from 100 m (Western Georgia) up to 900–1000 m a.s.l., reaching the absolute upper limit at 1400 m in sporadic locations of West and East Georgia [1,2]. According to the official International Union for Conservation of Nature (IUCN) list, *Castanea sativa* has been assessed as Least Concern [3,4]. Because of low self-renewal and pathogenic diseases, the large massifs of chestnut forests in Georgia are on the verge of destruction [5]. Therefore, sweet chestnut has been included in the Red List of Georgia under state Vulnerable (VU), according to the IUCN Red List Categories and Criteria [6,7]. The reason for including *Castanea sativa* in the Red List is the fragmentation and decreased distribution range. Hereafter, the development of efficient conservation measures is essential for both economic and wildlife protection commitments. 

The ex situ conservation of chestnut in seed banks is limited due to nonresistance to storage at low-temperature conditions of partially dehydrated recalcitrant seeds [8,9]. Medium- (by in vitro slow-growth storage) and long-term preservation techniques (by cryopreservation in liquid nitrogen (LN)) are currently widely used for selected germplasm collections of various woody perennials [10,11,12]. In chestnut, Janeiro et al. reported the successful medium-term preservation of chestnut hybrid clones since the mid-1990s [13]. *Castanea* shoot cultures remained viable between 5 and 18 months of slow-growth storage at 4–8 °C [14,15,16]. Depending on storage conditions, up to 82% of explants survived and resumed normal growth [17]. As for cryopreservation, this method has been broadly used for the preservation of different biological materials of chestnut species and hybrid clones, such as shoot tips [18,19], zygotic embryonic axes [8,20,21] and embryogenic cultures [22,23,24], where desiccation and Plant Vitrification Solution 2 (PVS2)-based vitrification techniques [25] have been practiced on naked explants. On the other hand, since its proposal in the early 1990s [26,27], the use of dehydrated encapsulated explants (generally named synthetic seeds) has become a valid alternative for the cryopreservation of many plant species [28,29]. Following the “encapsulation–dehydration” method, a new variant, termed “encapsulation–vitrification,” was proposed [27,30]. This method combines the advantages of the vitrification and encapsulation of explants, greatly reducing the time required to apply a protocol in comparison to the “encapsulation–dehydration” method. So far, the “encapsulation–vitrification” technique has successfully been applied to the cryopreservation of the shoot tips of several fruit crops [31,32,33] and embryogenic cell suspensions of grapevine (*Vitis* spp.) [34].

The present study describes, for the first time, a protocol for the cryopreservation of the excised embryonic axes (EAs) of *Castanea sativa* L. by using the “encapsulation–vitrification” approach to evaluate its effectiveness in long-term conservation. Furthermore, the addition of activated charcoal (AC) as a component of the artificial matrix of synthetic seeds to diminish the polyphenol toxicity was tested with the aim to facilitate the optimization of the tested new cryoprocedure.

## 2. Results

### Effect of LS and PVS2 Treatments on Survival and Regrowth of Encapsulated EAs

**Figure 1:**
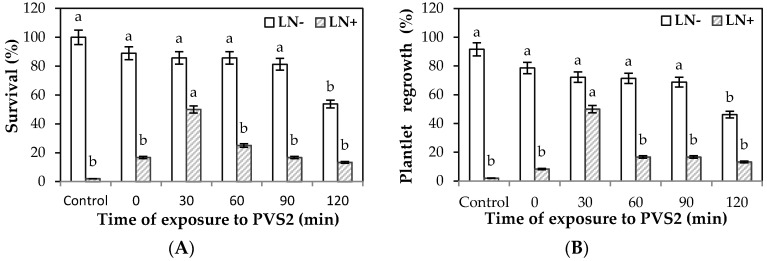
Percentages of survival (**A**) and plantlet regrowth (**B**) of encapsulated *Castanea sativa* embryonic axes (EAs) after exposure to Plant Vitrification Solution 2 (PVS2) for increasing times, with (LN+) or without (LN−) subsequent immersion in liquid nitrogen. EAs were encapsulated in WPM medium containing 2.5% (*w*/*v*) sodium alginate. Encapsulated EAs were treated for 60 min with a loading solution (2.0 M glycerol, 0.4 M sucrose), followed by treatment with PVS2 at 0 °C for 30–120 min, prior to direct immersion in LN for 1 h. Control EAs received no LS and PVS2 treatments. Within each line (LN− and LN+), data followed by different letters are significantly different at *p* ≤ 0.05 by LSD test (bars, SE of means).

Treatment of alginate-coated EAs with only a loading solution (LS, containing 2 M glycerol and 0.4 M sucrose) for 60 min at 25 °C induced a small reduction of survival from 100% (control, nontreated and noncryopreserved) to 88.9% (time 0); however, treatment with LS positively influenced the survival rate of noncryostored (LN−) encapsulated EAs after treatment with PVS2. EA survival remained between 85.7% (30 and 60 min of treatment) and 81.3% (90 min), with no significant differences in percentage values. Only the survival of EAs treated for 120 min was reduced to 53.8% (Figure 1A). Similar findings were observed with the regrowth rates of noncryopreserved encapsulated EAs. EA regrowth remained in the range of 72.2% to 68.8% (30 and 90 min of treatment), and the regrowth of EAs treated for 120 min was reduced to 46.2% (120 min) (Figure 1B). However, only the loading treatment induced tolerance to ultrarapid cooling in LN, as survival and plantlet regrowth of encapsulated EAs passed from nil to almost 16.7% and 8.3%, respectively (Figure 1A,B, 0 treatment).

The PVS2 treatment duration significantly affected the survival of cryopreserved (LN+) encapsulated EAs. The 30 min treatment with PVS2 induced the highest (50%) survival of EAs. A further increase in PVS2 treatment time to 120 min resulted in a significant decline of cryopreserved EA survival up to a minimum of 13.3% (Figure 1A). Referring to the regrowth of plantlets, derived from “germinated” synthetic seeds, noncryopreserved encapsulated EAs exhibited a decrease in regrowth, ranging from 91.6% in control plants to 46.2% after treatment with PVS2 at 120 min (Figure 1B). Plantlet regrowth rates were significantly reduced after cryogenic storage at –196 °C. The 30 min PVS2 treatment was the most effective in inducing tolerance to ultrarapid cooling in LN, resulting in 50% plantlet regrowth in postcryopreservation (Figure 1B). A further increase in PVS2 treatment time yielded a significant reduction of cryopreserved EA regrowth up to a minimum of 13.3% (Figure 1B).

**Figure 2:**
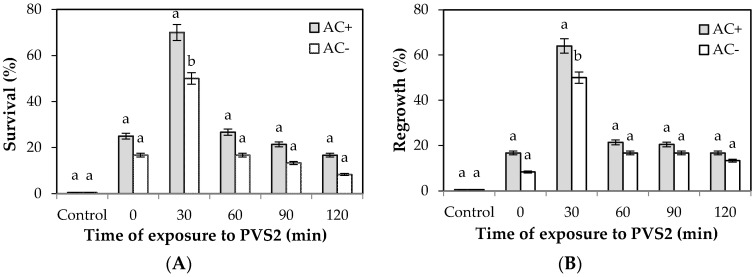
Effect of activated charcoal on the survival (**A**) and regrowth (**B**) of cryopreserved (LN+) chestnut embryonic axes by encapsulation–vitrification. EAs were encapsulated in WPM solution containing 2.5% (*w*/*v*) sodium alginate with (AC+) or without (AC−) 0.3% (*w*/*v*) activated charcoal in the artificial matrix. Encapsulated EAs were treated for 60 min with LS (2.0 M glycerol, 0.4 M sucrose), followed by treatment with PVS2 at 0 °C for 30–120 min, prior to direct immersion in liquid nitrogen for 1 h. Control EAs received no LS and PVS2 treatments. Within each exposure time, different letters indicate significant differences between AC+ and AC− at *p* ≤ 0.05 by chi-squared test (bars, SE of means).

Survival values were significantly different when EAs were encapsulated in alginate beads containing 0.3% activated charcoal (AC) in the artificial matrix. The best results were achieved after the 30 min treatment with PVS2 with a survival rate of 70% in cryopreserved EAs, significantly higher than in non-AC beads (50%; Figure 2A). 

The presence of AC significantly influenced regrowth rates in EAs. The regrowth of AC-encapsulated EAs after treatment with PVS2 for increasing times was in the range of 21.4% and 20.5% for 60 and 90 min of treatment, respectively, whereas it was 16.7% for 120 min of treatment, with no significant differences in percentage values (Figure 2B). In comparison, treatment with PVS2 for 30 min yielded 64% regrowth, showing to be the best recovery rate of AC-added cryopreserved encapsulated EAs, significantly higher than EAs encapsulated without AC (50%, Figure 2B). 

The shoot and root length data summarized in Table 1 also show the effect of AC on the plantlet regrowth of encapsulated EAs subjected to various vitrification times with PVS2, five weeks after cryostorage, rewarming and plating. 

**Figure 3:**
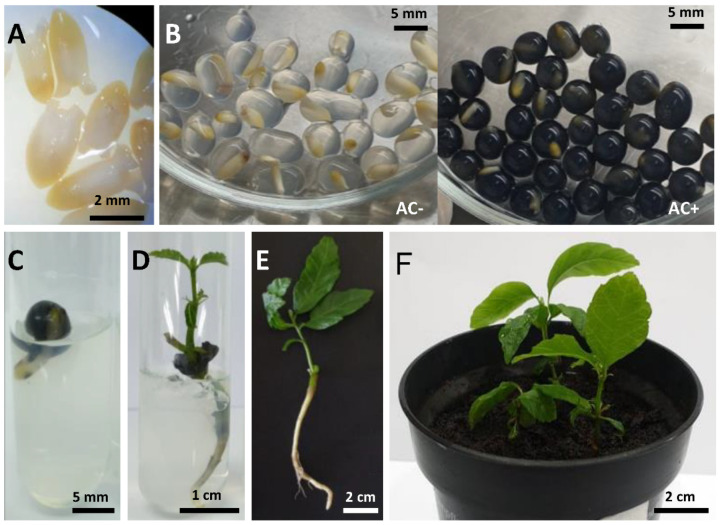
Plant regeneration from cryopreserved embryonic axes of *Castanea sativa* by encapsulation–vitrification. (**A**) Excised embryonic axes (EAs) used for cryopreservation. (**B**) Encapsulated EAs in 2.5% sodium alginate with (AC+) or without (AC−) 0.3% (*w*/*v*) activated charcoal in the artificial matrix. (**C**) Survived encapsulated (AC+) EAs after cryopreservation and 20 days of postculture. (**D**) Primary plantlet development 2 weeks after survival assessment cryopreserved by encapsulation–vitrification procedure. (**E**) Elongated root and shoot 8 weeks of postculture after cryopreservation. (**F**) Plantlets established under greenhouse conditions 4 weeks after transfer to soil.

All surviving cryostored EAs produced roots and shoots, and their development was clearly pronounced in both (AC−) and (AC+) synthetic seeds with the 30 min PVS2 treatment time (Table 1). Moreover, it is noteworthy that with AC added to the synthetic seed, an appreciable shoot and root length was highlighted during postcryopreservation (Figure 3D), with 14.5 and 22.8 cm, respectively, after five weeks, whereas without AC, the cryopreserved plantlets had 9.2 mm and 10.2 mm of the shoot and root length at the same period. The germination of noncryopreserved synthetic seeds started after one week of culture, and it was delayed up to four weeks after cryogenic storage in LN. However, the regrowth initiation time was shorter in encapsulated explants containing AC, i.e., 20 days in postcryopreservation (Figure 3C). After eight weeks of culture, all (AC+)-derived plantlets showed well-developed roots and shoots that allowed their transfer in greenhouse conditions (Figure 3D–F).

## 3. Discussion

Synthetic seed technology, in addition to fulfilling needs related to micropropagation [35,36,37,38,39], can prove to be an efficient tool for the storage of rare and commercially important species at low temperatures. It has the potential for the medium-term and long-term preservation of plant explants encapsulated in synthetic seeds, without losing viability after immersion in LN when cryopreservation is applied [26,27,28,40,41].

The “encapsulation–vitrification” cryoprocedure [27] has been used for the cryopreservation of the shoot tips of several woody fruit crops [28,31,32,33] and embryogenic cell suspensions [34]. Although it requires a long treatment time compared to the vitrification of naked explants, the encapsulation of plant germplasm makes for less damage to samples during the vitrification procedures [42,43]. In our encapsulation–vitrification experiment with chestnut EAs, after treatment with LS for 60 min, the 30 min exposure time of PVS2 showed the best regrowth rate (50%). Optimizing the time of exposure to PVS2 is most important for producing a satisfactory level of regrowth after cryopreservation, and the PVS2 osmoprotection effect can change among different species [28,44]. For example, the duration of PVS2 treatment was up to 200 min in the encapsulated shoot tips of *Dianthus caryophyllus* L [45]. 

In the following experiment, the addition of AC into synthetic seeds treated with the same conditions positively affected plantlet initiation and regrowth from chestnut EAs, with the concentration amended with 0.3% (*w*/*v*) AC. In a previous research, AC was added in a culture medium to overcome the onset of browning, shortly after the excision of EAs, and promoted their germination [21]. In another study, AC added in the artificial endosperm of synthetic seeds containing somatic embryos of hybrid rice improved their germination and conversion to plantlets [46]. Furthermore, the germination and root development of encapsulated somatic embryos *of Picea glauca* and *Picea mariana* enhanced with the addition of 0.05 gL^−1^ AC to the beads [47]. Therefore, as also shown in this study, AC represents a component that can improve the development of explants even after their ultrarapid cooling in LN. Indeed, the survival and regrowth rates of cryopreserved encapsulated EAs were markedly increased when AC was included in the bead composition. 

It is also noteworthy that the results obtained here showed an improvement in the survival and regrowth of cryostored chestnut EAs by 15% and 10%, respectively, in comparison with a previous study concerning the vitrification procedure of naked EAs [21]. Although the survival and regrowth rates of encapsulated (AC−) AEs were lower than the same parameters obtained in a previous study by the “desiccation–one-step cooling” protocol (70% and 64%, respectively) [21], it should be noted that the overall ratio between embryo survival and plantlet regrowth appreciably improved with the presence of AC in synthetic seeds. Thus, the survival/regrowth ratio of AC-encapsulated AEs after cryopreservation was 91.4% versus 83% obtained by the “desiccation–one-step freezing procedure,” at the best treatment times. Corredoira et al., [20] reported a 63.3% recovery applied to *Castanea sativa* zygotic embryos by the desiccation procedure, which was still lower than the regrowth percentage obtained in our experiment with (AC+) encapsulation–vitrification. 

The conversion of synthetic seeds into plants after germination is a fundamental aspect of the success of the encapsulation–vitrification technique. In this study, the development of cryopreserved encapsulated EAs with (AC+), after treatment with 30 min PVS2, was faster by 6–7 days with respect to EAs encapsulated without AC. Furthermore, the root and shoot length of (AC+) EAs achieved 22.8 mm and 14.5 mm, respectively, five weeks after rewarming and plating, whereas the (AC−) EAs showed less development at the same period (Table 1). Evident differences were also found in the postcryopreservation initiation times of plantlet formation with respect to previous cryopreservation procedures applied on EAs. Indeed, the plantlet development of cryopreserved EAs synthetic seeds with (AC+) started two weeks earlier than naked vitrified EAs, where the full germination of EAs (expressed as plantlet regrowth) required eight weeks and eight days earlier than desiccated by dehydration–”one-step freezing” EAs [21]. Notably, even root and shoot elongation from encapsulated (AC+) EAs was considerably faster, exceeding 2 cm root and 1.5 cm shoot length in five weeks.

AC seems to play a role to keep nutrients within the artificial matrix, releasing them slowly during the development of embryos. The absorption of detrimental polyphenolic exudates released by encapsulated explants is also facilitated by AC [48]. 

## 4. Materials and Methods

### 4.1. Plant Material

Chestnut fruits were collected in Western Georgia at the beginning of October 2019 from the open-pollinated trees of *Castanea sativa*. Mature fruits were stored at 4 °C for a maximum of 1 month until use in the cryopreservation trials. For the cryopreservation experiments, the fruits were washed in 2% (*v*/*v*) household detergent and rinsed three times under tap water. Then, the pericarp, seed coat and part of the kernel were removed. The remaining embryo axes along with the part of the kernel were surface-sterilized by successive immersion in 70% (*v*/*v*) ethanol with a few drops of Tween 20 for 2 min, followed by decontamination with a 10% (*v*/*w*) solution of sodium hypochlorite (Sigma-Aldrich^®^, Saint Louis, MO, USA) for 20 min. After being rinsed in sterile distilled water three times, EAs, composed of the zygotic embryos along with 2–3 mm long cotyledon residuals, were dissected from the seeds (Figure 3A). 

### 4.2. Encapsulation

Dissected EAs were immersed in a calcium-free liquid woody plant medium (WPM) [49] without plant growth regulators, supplemented with 2.5% (*w*/*v*) sodium alginate (Bioworld^®^, Dublin, OH, USA) and 0.3 M sucrose. The mixture (including dissected EAs) was dropped with a sterile pipette into WPM liquid medium containing 100 mM calcium chloride, forming beads about 4–5 mm in diameter (Figure 3B). The drops with EAs were maintained in the solution for 20 min to achieve polymerization. 

In one specific experiment, 0.3% (*w*/*v*) activated charcoal (AC; Sigma-Aldrich, DARCO^®^, Saint Louis, MO, USA) was added to the sodium alginate solution to assess its influence on the survival and regrowth rate of encapsulated EAs after PVS2 treatment and subsequent cooling. All operations were performed under sterile conditions. After the incubation period in the complexion agent, the encapsulated explants were rinsed three times in sterile distilled water.

### 4.3. Encapsulation–Vitrification Technique for EA Cryopreservation

Encapsulated EAs were transferred to LS containing 2.0 M glycerol and 0.4 M sucrose for 60 min at 25 °C, followed by treatment with PVS2 [25] (30% *w*/*v* glycerol, 15%, *w*/*v* DMSO, 15% *w*/*v* ethylene glycol in WPM medium containing 0.4 M sucrose) for 0, 30, 60, 90, 120 min treatment times at 0 °C. Then, synthetic seeds were placed in 2 mL cryovials (5 in each) and immersed in LN for 24 h (LN+). For rewarming, the cryovials were rapidly immersed in a water bath at 40 °C for 2 min. Encapsulated EAs were rinsed in a washing solution containing the WPM liquid medium and 1.2 M sucrose (two times of 10 min each, at 25 °C), and then LN+ and LN− (synthetic seeds without cooling) samples were cultured in test tubes (20 mm × 150 mm) in WPM supplemented with 30 g L^−1^ sucrose and 0.4 µM 6-benzylaminopurine (BAP). The medium was solidified with 6 g L^−1^ agar (PlantMedia™, Dublin, OH, USA) and adjusted to pH 5.7 before autoclaving. Cultured tubes were maintained in a growth chamber at 24 ± 0.5 °C under a 16/8 h light/dark regime with an irradiance of 40 μmol m^−2^ s^−1^ in cool-white fluorescent light. After 2 and 4 weeks, survival and regrowth were recorded, respectively. After 8 weeks of in vitro culture, plantlets (i.e., “germinated” EAs) were washed thoroughly in running tap water; the root length was measured and transferred to plastic cups filled with a mixture of 100% sphagnum peat/perlite at a ratio of 2:1. The plantlets were relocated for acclimatization in controlled chambers at 23 ± 1 °C under 60 ± 5% moisture content and 16 h photoperiod with an irradiance of 40 μmol m^−2^ s^−1^ in cool-white fluorescent light over the following three weeks. After the emergence of new leaves, the plants were transplanted in bigger pots containing peat, soil and perlite at a ratio of 1:2:1 and transferred to natural greenhouse conditions.

### 4.4. Data Collection and Statistical Analysis

The total number of embryos used for the experiment was 240. Each treatment, with (AC+) or without (AC−)-activated charcoal, included 50 noncryopreserved (LN−) encapsulated EAs (10 EAs for each condition from 0 to 120 min treatment time) and 50 cryopreserved (LN+) encapsulated EAs (10 EAs for each condition). Control EAs (20 for each, AC− and AC+ treatments), receiving no LS, PVS2 or LN treatment, were also included. For a 0 h-min PVS2 treatment time, encapsulated EAs were only loaded in LS solution and cryopreserved without PVS2 or directly cultured in test tubes for synthetic seed “germination.” Each treatment consisted of 3 replicates, and all experiments were repeated 3 times.

Survival was recorded after two weeks of culture and defined as the percentage of the total number of encapsulated EAs, which showed initial normal germination and development (i.e., root and shoot emission) or only root development. The regrowth of encapsulated EAs was assessed after four weeks of culture. Plant regrowth rate was estimated as a percentage of whole plantlets (retaining normal shoots and roots ≥5 mm in length) developing from encapsulated EAs relative to the total number of synthetic seeds cultured after cryopreservation. Root and shoot length were recorded weekly. Statistical analysis of percentages was performed by ANOVA (when comparing multiple treatments), followed by the LSD test at *p* ≤ 0.05 for mean separation or chi-squared test (when comparing pairs of treatments). Percentage data used in ANOVA were subjected to arcsine transformation prior to analysis. The bars in the figures represent standard errors (SE) of means. 

## 5. Conclusions

The present study has clearly demonstrated the feasibility of the long-term preservation of *Castanea sativa* germplasm by the encapsulation–vitrification of EAs. The acquisition of suitable dehydration tolerance with PVS2 to survive after the cryopreservation of EA synthetic seeds and their germination and regrowth under optimized conditions (AC+) promoted growth by shortening the development times and limiting the loss of explants; therefore, the overall performance of the cryopreserved EAs appears to be improved in comparison with previous studies. The protocol described in this study will now be tested on a wide range of chestnut cultivars and hybrid clones to achieve the practical long-term cryopreservation of *Castanea* genus germplasm.

## Figures and Tables

**Table 1:** Effect of activated charcoal (AC) on the plantlet regrowth of encapsulated *Castanea sativa* EAs subjected to various dehydration times with PVS2 following immersion in LN evaluated 5 weeks after cryostorage, rewarming and plating.

PVS2 (min)	Regrowth ^a^ (AC−)		Regrowth ^a^ (AC+)	
	Shoot Length (mm)	Root Length (mm)	Shoot Length (mm)	Root Length (mm)
0	4.0 b	5.2 b	5.0 c	6.3 c
30	9.2 a	10.2 a	14.5 a	22.8 a
60	6.5 b	8.8 a	8.7 b	9.8 b
90	5.9 b	8.9 a	8.3 b	10.4 b
120	4.9 b	7.8 a	7.9 b	9.5 b

^a^ Mean of 90 plantlets tested. Data were recorded after 5 weeks of culture following cryopreservation. Statistical analysis in each column was performed by ANOVA. Data followed by different letters are significantly different at *p* ≤ 0.05 by LSD test.
